# Supplementary material for: Mechanism of SARS-CoV-2 resistance to nucleotide analog-based antivirals
Source: Nat Commun. 2026 Jan 13;17:1601. doi: 10.1038/s41467-026-68304-8 (PMC12905345; doi:10.1038/s41467-026-68304-8)
Supplement: Supplementary file 1 — Supplementary Information [file 41467_2026_68304_MOESM1_ESM.pdf]

Supplementary Information for

Mechanism of SARS-CoV-2 resistance to nucleotide analog-based antivirals

Chang Liu[1,*], Yu Li[2], Xiaocong Cao[1], Ryan J. Gleason[1], Bin Liu[3,*], Yang Yang[2,*]

*Corresponding author. Email: yan9yang@iastate.edu (Y.Y.); liu00794@umn.edu (B.L.); cliu207@jhmi.edu (C.L.)

The PDF file includes:

Supplementary Figures 1–9

Supplementary Tables 1–2

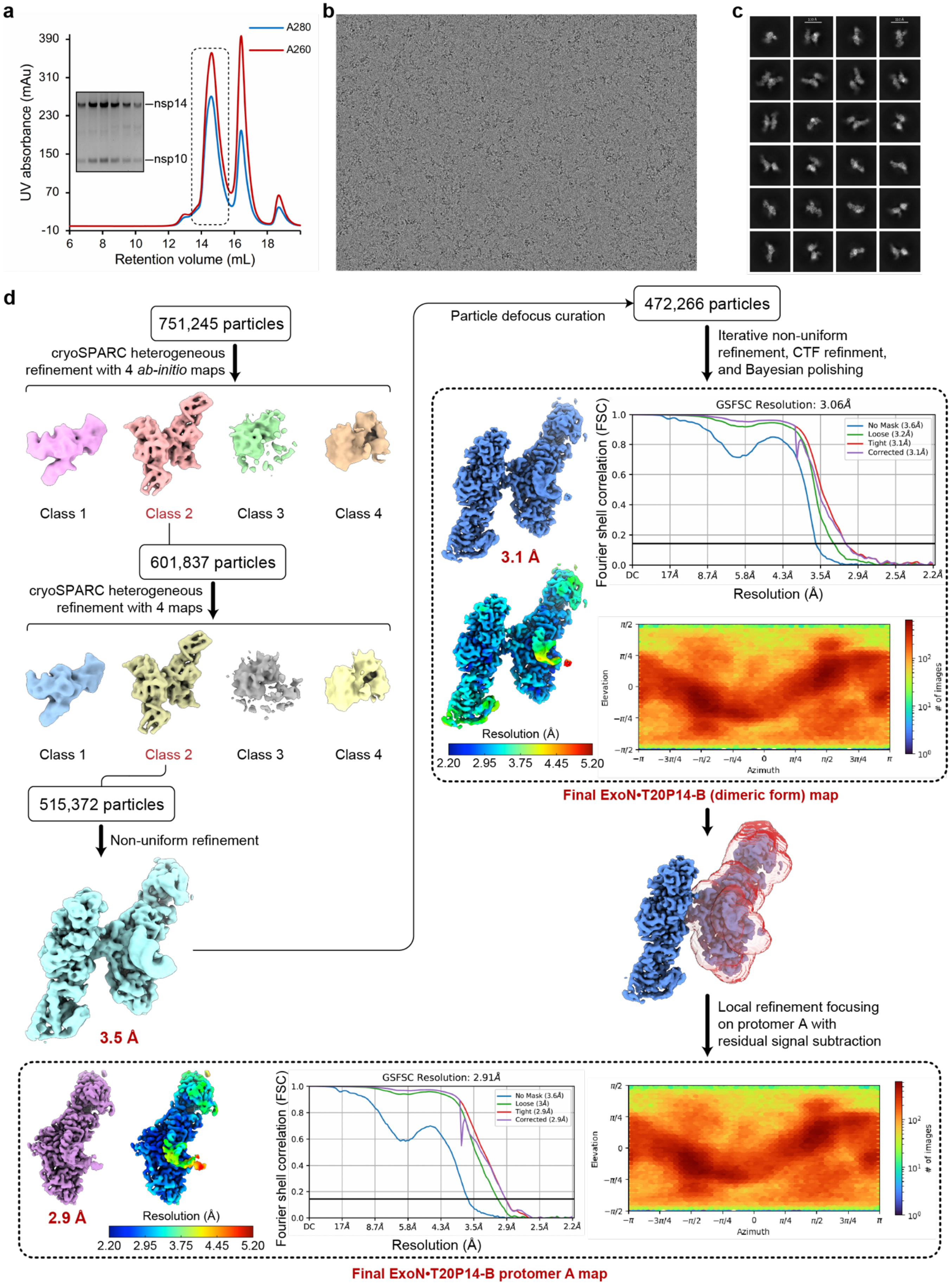

a
A280
A260
UV absorbance (mAu)
Retention volume (mL)
nsp14
nsp10
b
c
d
751,245 particles
cryoSPARC heterogeneous refinement with 4 ab-initio maps
Class 1
Class 2
Class 3
Class 4
601,837 particles
cryoSPARC heterogeneous refinement with 4 maps
Class 1
Class 2
Class 3
Class 4
515,372 particles
Non-uniform refinement
3.5 Å
Particle defocus curation
472,266 particles
Iterative non-uniform refinement, CTF refinment, and Bayesian polishing
3.1 Å
GSFSC Resolution: 3.06Å
No Mask (3.6Å)
Loose (3.2Å)
Tight (3.1Å)
Corrected (3.1Å)
Fourier shell correlation (FSC)
Resolution (Å)
Elevation
Azimuth
# of images
Final ExoN•T20P14-B (dimeric form) map
Local refinement focusing on protomer A with residual signal subtraction
2.9 Å
GSFSC Resolution: 2.91Å
No Mask (3.6Å)
Loose (3Å)
Tight (2.9Å)
Corrected (2.9Å)
Final ExoN•T20P14-B protomer A map

Supplementary Fig. 1: Single-particle cryo-EM analysis of SARS-CoV-2 ExoN•T20P14-B complex. (**a**) SEC purification of SARS-CoV-2 ExoN•T20P14-B complex. The protein compositions in the dashed line boxed fractions were analyzed by SDS-PAGE and are shown in the embedded gel panel. (**b**) A representative micrography from the ExoN•T20P14-B complex. (**c**) Representative 2D classes generated from the SARS-CoV-2 ExoN•T20P14-B complex cryo-EM dataset. (**d**) Flow chart of cryo-EM image processing and map reconstruction for the ExoN•T20P14-B complex. The final cryo-EM maps of the complex, local resolution illustrations, the accompanying half-map FSC plots, and the angular distribution plots of particles used in the final reconstructions are enclosed in the dashed boxes. Source data are provided as a Source Data file.

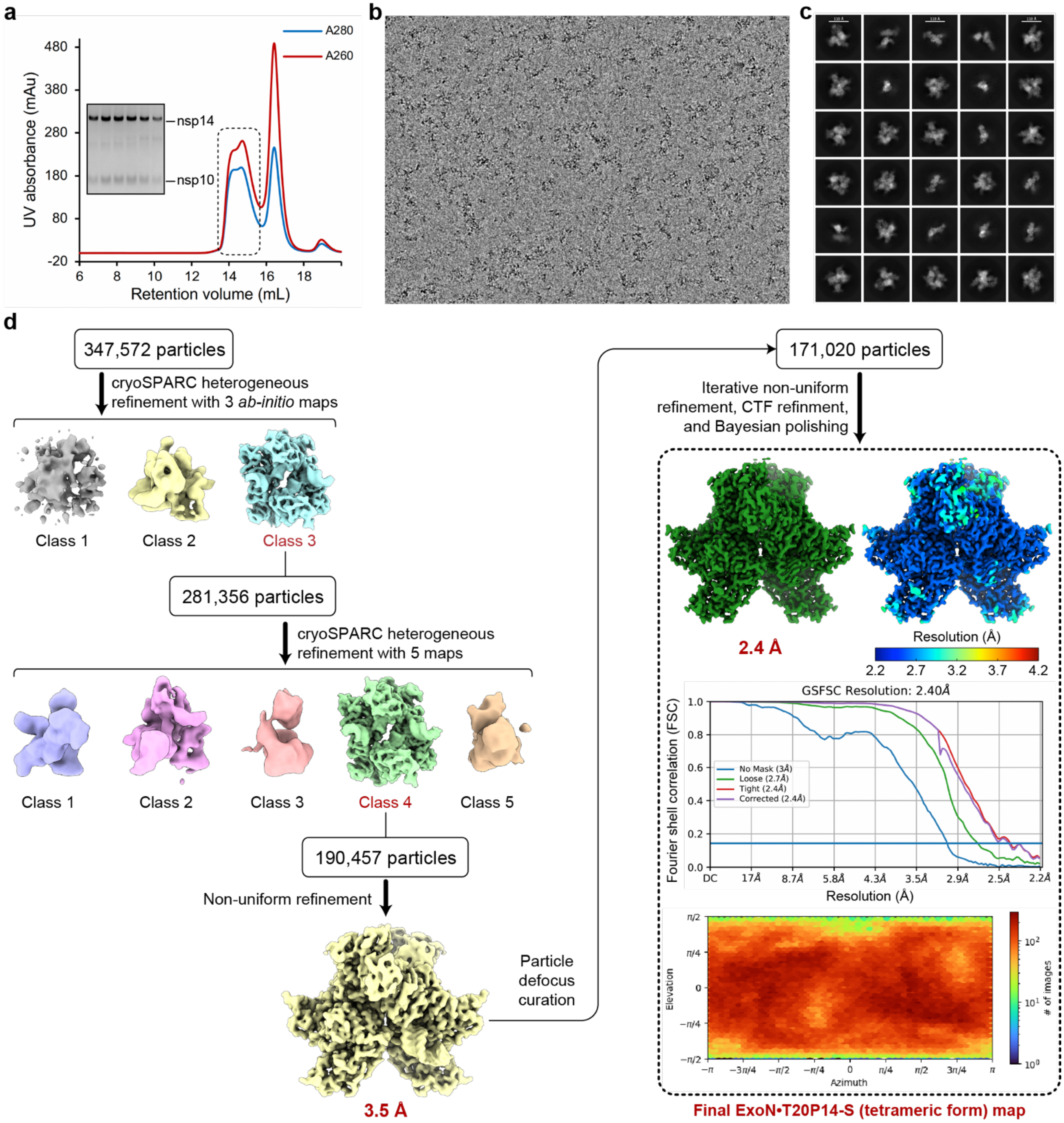

Supplementary Fig. 2: Single-particle cryo-EM analysis of the tetrameric form of the SARS-CoV-2 ExoN•T20P14-S complex. (**a**) SEC purification of SARS-CoV-2 ExoN•T20P14-S complex. The protein compositions in the dotted-line boxed fractions were analyzed by SDS-PAGE and are shown in the embedded gel panel. (**b**) A representative micrograph from the tetrameric form of the ExoN•T20P14-S complex dataset. (**c**) Representative 2D classes generated from the tetrameric form of the ExoN•T20P14-S complex cryo-EM dataset. (**d**) Flow chart of cryo-EM image processing and map reconstruction for the tetrameric form of the ExoN•T20P14-S complex. The final cryo-EM map of the complex, the local resolution illustration, the accompanying half-map FSC plots, and

the angular distribution plot of particles used in the final reconstruction are enclosed in the dashed box. Source data are provided as a Source Data file.

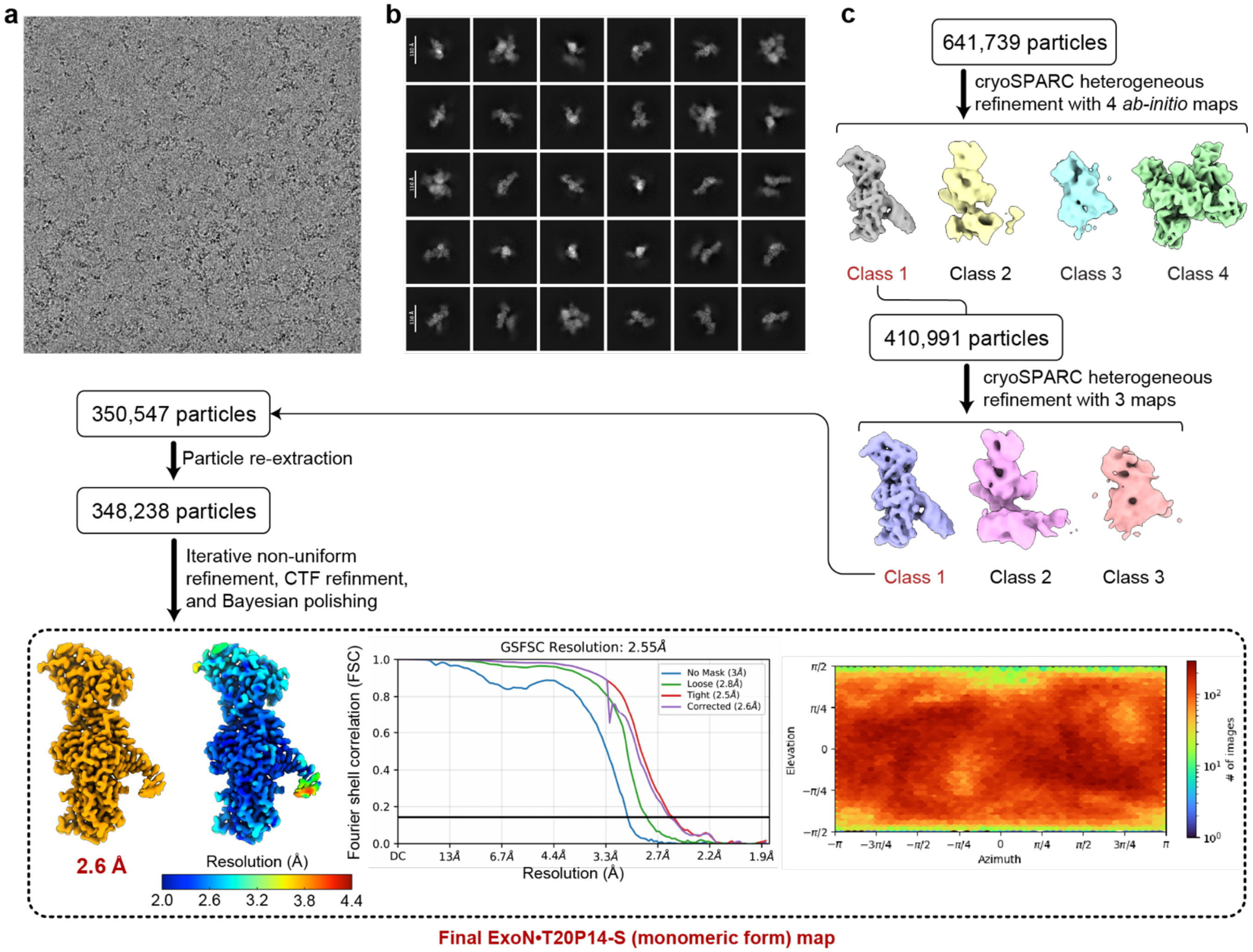

Supplementary Fig. 3: Single-particle cryo-EM analysis of monomeric form of the SARS-CoV-2 ExoN•T20P14-S complex. (**a**) A representative micrograph from the monomeric form of the ExoN•T20P14-S complex dataset. (**b**) Representative 2D classes generated from the monomeric form of the ExoN•T20P14-S complex cryo-EM dataset. (**c**) Flow chart of cryo-EM image processing and map reconstruction for the monomeric form of the ExoN•T20P14-S complex. The final cryo-EM map of the complex, the local resolution illustration, the accompanying half-map FSC plots, and the angular distribution plot of particles used in the final reconstruction are enclosed in the dashed box.

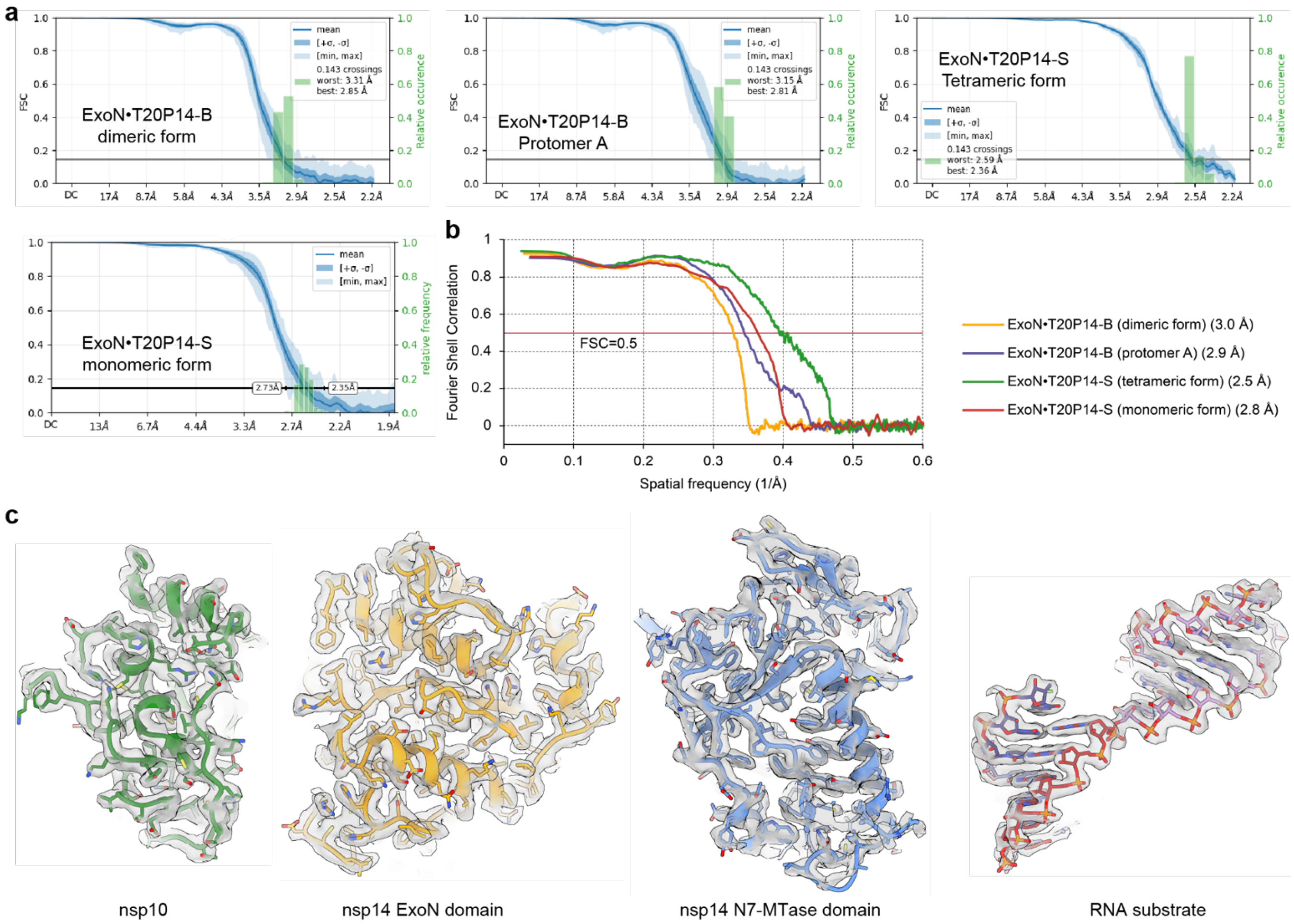

Supplementary Fig. 4: Validation of cryo-EM maps and models. (**a**) Orientation diagnosis and histograms of 3D FSC plots for the cryo-EM maps. (**b**) Model-map FSC curves of the four atomic structures and their corresponding cryo-EM maps from this study were generated from Phenix comprehensive validation results. The model-map resolution for each structure at FSC = 0.5 cutoff is indicated in the figure and summarized in Supplementary Table 1. (**c**) Cryo-EM densities superimposed on a structural model of representative regions of SARS-CoV-2 ExoN•RNA complexes determined in this study. Cryo-EM densities are contoured at 4σ and shown as gray surfaces. Source data are provided as a Source Data file.

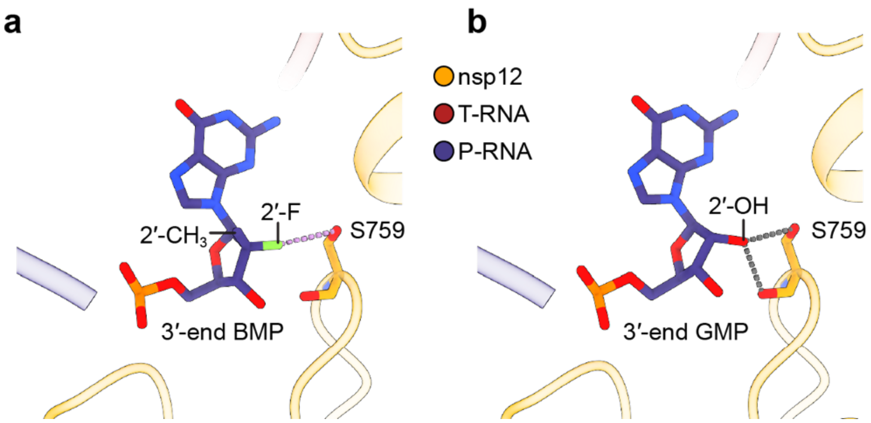

Supplementary Fig. 5: Structural basis of weakened interactions between SARS-CoV-2 RdRp and bemnifosbuvir- and sofosbuvir-incorporated RNAs. (**a**) A weak hydrogen bond formed between nsp12 S759 and the 2'-fluoro group of the RNA 3'-end BMP. The structural illustration was prepared using PDB 7ED5. The weak fluoro-hydroxyl hydrogen bond is shown as a pink dashed line. (**b**) A favorable bi-furcated hydrogen bond between nsp12 S759 and the 2'-OH group of a modeled RNA 3'-end GMP. Hydrogen bonds are shown as gray dashed lines.

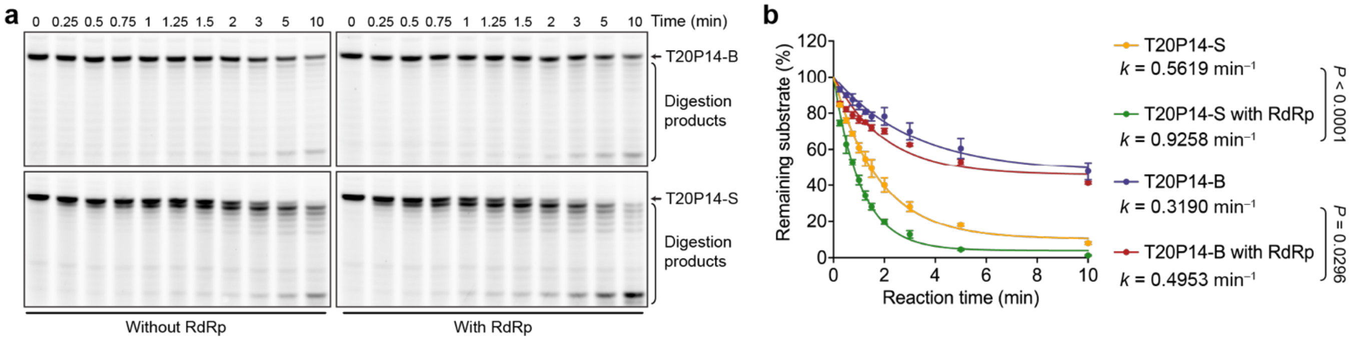

Supplementary Fig. 6: RdRp enhances ExoN-mediated excision of bemnifosbuvir and sofosbuvir from RNA. (**a**) Digestion of T20P14-S or T20P14-B by SARS-CoV-2 ExoN in the absence or presence of RdRp. The exonucleolytic digestion reactions were stopped at the indicated time points. The RNA products were resolved by denaturing PAGE and visualized by FAM imaging. A representative result from three biological replicates is shown. (**b**) Percentages of substrate RNAs remaining shown in (**a**) were quantified using Bio-Rad Image Lab from three independent experiments and are shown as mean ± SEM. The results were plotted in GraphPad Prism using the One-phase decay model. The decay rate constant (k) for each reaction is indicated. Statistical analyses were performed using the two-sided extra sum-of-squares F test. P values for the comparisons of decay rate constants are indicated. Source data are provided as a Source Data file.

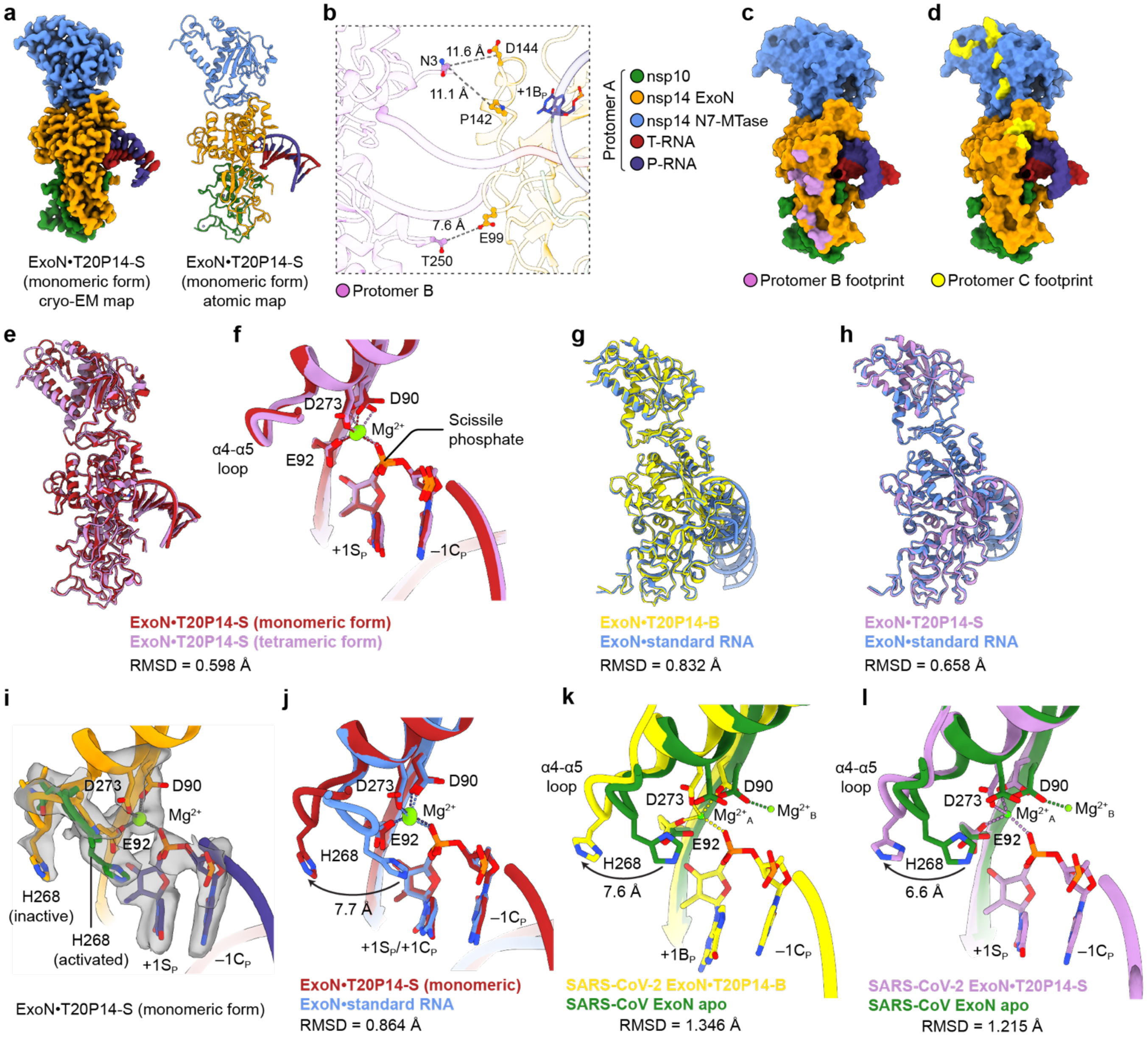

Supplementary Fig. 7: Structural analyses of SARS-CoV-2 ExoN•T20P14-B and SARS-CoV-2 ExoN•T20P14-S complexes. (**a**) Cryo-EM map and atomic model of SARS-CoV-2 ExoN•T20P14-S complex (monomeric form). (**b**) In the dimeric form of the SARS-CoV-2 ExoN•T20P14-B complex, residues surrounding the ExoN active site of each protomer are not involved in inter-protomer interactions. The shortest distances between ExoN residues in protomer A and protomer B are indicated. (**c**) Footprint of protomer B (colored in purple) shown on the surface representation of protomer A in the tetrameric form of ExoN•T20P14-S complex. (**d**) Footprint of protomer C (colored in yellow) shown on the surface representation of protomer A in the tetrameric form of ExoN•T20P14-S complex. (**e,f**) Superimposition of the (**e**) overall structure and (**f**) ExoN active site conformation between the monomeric and tetrameric forms of the SARS-CoV-2 ExoN•T20P14-S complex. RMSD of the overall structure superimposition is indicated. (**g**) Superimposition of the SARS-CoV-2 ExoN•T20P14-B complex and the ExoN•standard RNA complex (PDB 7N0C). RMSD of the superimposition is indicated. (**h**) Superimposition of the SARS-CoV-2 ExoN•T20P14-S complex

and the ExoN•standard RNA complex (PDB 7N0D). RMSD of the superimposition is indicated. (**i**) ExoN active site conformation of the SARS-CoV-2 ExoN•T20P14-S complex (monomeric form). $+1S_P$, $–1C_P$, Mg^{2+} ion, three catalytic carboxylate residues, and nsp14 α4-α5 loop are superimposed with their cryo-EM densities. The inactive and activated conformations of the α4-α5 loop are colored in orange and green, respectively. (**f**) Superimposition of ExoN active site in the presence of T20P14-S (monomeric form) or a standard RNA (PDB ID 7N0C). For clarity, only the inactive conformation of the nsp14 α4-α5 loop in the ExoN•T20P14-S complex is shown. RMSD of the superimposition is indicated. (**k**) Superimposition of ExoN active sites in the SARS-CoV-2 ExoN•T20P14-B complex and the apo SARS-CoV ExoN complex (PDB 5C8U). Mg^{2+} ions, green spheres. Metal coordination bonds are shown as dashed lines. (**l**) Superimposition of ExoN active sites in the SARS-CoV-2 ExoN•T20P14-S complex and the apo SARS-CoV ExoN complex (PDB 5C8U). For clarity, only the inactive conformation of the nsp14 α4-α5 loop in the ExoN•T20P14-S complex is shown.

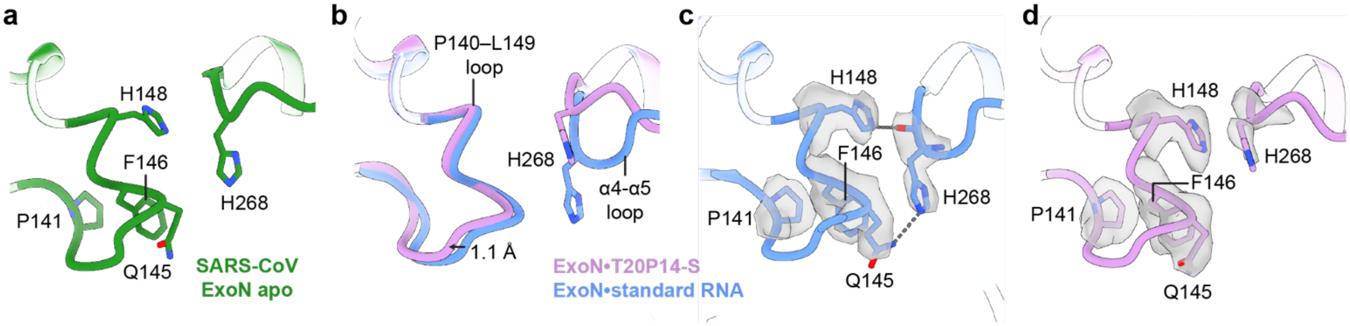

Supplementary Fig. 8: Allosteric regulation of ExoN active site assembly. (**a**) Absence of bridging interactions between the nsp14 P140–L149 loop and α4-α5 loop in the ExoN apo structure (PDB 5C8U). (**b**) Superimposition of nsp14 P140–L149 loop and α4-α5 loop between ExoN•T20P14-S (protomer A) and ExoN•standard RNA complexes. The displacement of the P140–L149 loop upon the binding of T20P14-S RNA is indicated. (**c**) Bridging interactions between the nsp14 P140–L149 loop and α4-α5 loop in the ExoN•standard RNA complex. Hydrogen bonds are shown as gray dashed lines. Nsp14 residues are superimposed with their cryo-EM densities. (**d**) Loss of bridging interactions between the nsp14 P140–L149 loop and α4-α5 loop in the ExoN•T20P14-S complex (protomer A). Nsp14 residues are superimposed with their cryo-EM densities.

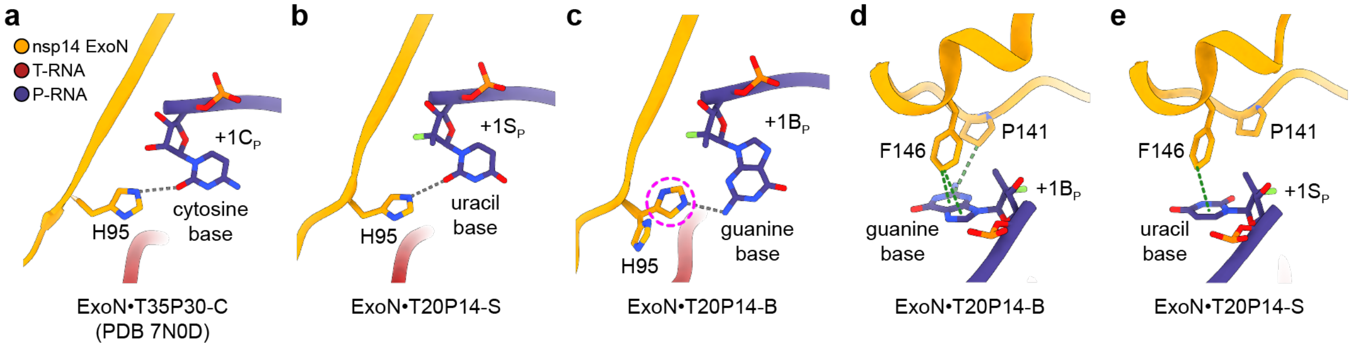

Supplementary Fig. 9: Nucleobase recognition by SARS-CoV-2 ExoN. (**a**) Interaction between SARS-CoV-2 ExoN and RNA 3'-end cytosine base. Hydrogen bond is shown as a dark gray dashed line. (**b**) Interaction between SARS-CoV-2 ExoN and the uracil base of the RNA 3'-end SMP. (**c**) Interaction between SARS-CoV-2 ExoN and the guanine base of the RNA 3'-end BMP. The distinct rotamer conformation of H95 in the ExoN•T20P14-B complex is highlighted in a dashed magenta circle. (**d**) Detailed interactions between P141 and F146 of ExoN and the guanine base of 3'-end BMP. Hydrophobic and stacking interactions are shown as green dashed lines. (**e**) Detailed interactions between P141 and F146 of ExoN and the uracil base of 3'-end SMP.

Supplementary Table 1: Cryo-EM data collection, refinement, and validation statistics.

	SARS-CoV-2 ExoN•T20P14-B Dimeric form (EMDB-73369) (PDB 9YRK)	SARS-CoV-2 ExoN•T20P14-B Protomer A (EMDB-73370) (PDB 9YRL)	SARS-CoV-2 ExoN•T20P14-S Tetrameric form (EMDB-73371) (PDB 9YRN)	SARS-CoV-2 ExoN•T20P14-S Monomeric form (EMDB-73372) (PDB 9YRO)
**Data collection and processing**				
Magnification	81,000	81,000	81,000	130,000
Voltage (kV)	300	300	300	300
Detector	Gatan K3	Gatan K3	Gatan K3	Falcon 4i
Electron exposure ($e^{-}/Å^2$)	53.33	53.33	53.13	42.51
Defocus range (μm)	1.0–2.0	1.0–2.0	1.0–2.0	1.0–2.0
Pixel size (Å)	1.0724	1.0724	1.0724	0.9254
Symmetry imposed	*C*1	*C*1	*C*2	*C*1
Initial particle images (no.)	751,245	751,245	347,572	641,739
Final particle images (no.)	472,266	472,266	171,020	348,238
Map resolution (Å)	3.1	2.9	2.4	2.6
FSC threshold	0.143	0.143	0.143	0.143
Map resolution range (Å)	2.2–5.2	2.2–5.2	2.2–4.2	2.0–4.4
**Refinement**				
Initial model used (PDB code)	7N0C	7N0C	7N0D	7N0C
Model resolution (Å)	3.0	2.9	2.5	2.8
FSC threshold	0.5	0.5	0.5	0.5
Model resolution range (Å)	34.9–2.8	27.0–2.3	40.3–1.8	26.4–2.5
Map sharpening *B* factor ($Å^2$)	115.8	90.9	71.6	110.7
Model composition				
Non-hydrogen atoms	10957	5811	21420	5441
Protein residues	1284	642	2572	641
Nucleotides	40	35	56	16
Ligands	13	7	28	7
B factors ($Å^2$)				
Protein	75.96	72.13	63.59	52.62
Nucleic acid	135.17	136.23	73.28	68.84
Ligand	79.14	59.35	60.85	62.20
R.m.s. deviations				
Bond lengths (Å)	0.004	0.005	0.006	0.005
Bond angles (°)	0.948	0.973	1.108	0.976
Validation				
MolProbity score	1.22	1.33	1.11	1.07
Clashscore	2.56	3.71	2.00	2.19
Poor rotamers (%)	0.00	0.00	0.00	0.00
Ramachandran plot				
Favored (%)	96.93	97.01	97.21	97.64
Allowed (%)	3.07	2.99	2.79	2.36
Disallowed (%)	0.00	0.00	0.00	0.00

Supplementary Table 2: RNA constructs used in this study

Name	Sequence (5′ to 3′)	Modifications	Source	Application
T20P14-B	GGGAACGGGAUUUUAAUAGC UUCGGCUAUUAAAAUCCCB	5′-triphosphate; 3′ bemnifosbuvir monophosphate	*In vitro* transcription and incorporation	Cryo-EM analysis
T20P14-S	GGGACAGGGAUUUUAAUAGC UUCGGCUAUUAAAAUCCC	5′-triphosphate; 3′ sofosbuvir monophosphate	*In vitro* transcription and incorporation	Cryo-EM analysis
FAM-T20P14-G	AGGAACGGGAUUUUAAUAGC UUCGGCUAUUAAAAUCCCG	5′ 6-FAM	IDT	Fluorescence polarization assay (Figs. 1d and 2a); Exoribonuclease assay (Figs. 2b, c and 5d); RdRp stalling rescue assay (Fig. 2d)
FAM-T20P14-B	AGGAACGGGAUUUUAAUAGC UUCGGCUAUUAAAAUCCCB	5′ 6-FAM; 3′ bemnifosbuvir monophosphate	IDT, followed by *in vitro* incorporation of bemnifosbuvir monophosphate	Fluorescence polarization assay (Figs. 1d and 2a); Exoribonuclease assay (Figs. 2b, c and 4f and Supplementary Fig. 6a, b); RdRp stalling rescue assay (Fig. 2d)
FAM-T20P14-U	AGGACAGGGAUUUUAAUAGC UUCGGCUAUUAAAAUCCCU	5′ 6-FAM	IDT	Fluorescence polarization assay (Figs. 1d and 2a); Exoribonuclease assay (Figs. 2b, c and 5b); RdRp stalling rescue assay (Fig. 2e)
FAM-T20P14-S	AGGACAGGGAUUUUAAUAGC UUCGGCUAUUAAAAUCCCS	5′ 6-FAM; 3′ sofosbuvir monophosphate	IDT, followed by *in vitro* incorporation of sofosbuvir monophosphate	Fluorescence polarization assay (Figs. 1d and 2a); Exoribonuclease assay (Figs. 2b, c and 4g and Supplementary Fig. 6a, b); RdRp stalling rescue assay (Fig. 2e)
FAM-T20P14-A	AGGAAUGGGAUUUUAAUAGC UUCGGCUAUUAAAAUCCCA	5′ 6-FAM	IDT	Exoribonuclease assay (Fig. 5a)
FAM-T20P14-C	AGGACGGGAAUUUUAAUAGC UUCGGCUAUUAAAAUUCCC	5′ 6-FAM	IDT	Exoribonuclease assay (Fig. 5c)
